# Spending at least 120 minutes a week in nature is associated with good health and wellbeing

**DOI:** 10.1038/s41598-019-44097-3

**Published:** 2019-06-13

**Authors:** Mathew P. White, Ian Alcock, James Grellier, Benedict W. Wheeler, Terry Hartig, Sara L. Warber, Angie Bone, Michael H. Depledge, Lora E. Fleming

**Affiliations:** 1 European Centre for Environment and Human Health, University of Exeter Medical School, Exeter, UK; 2 Institute for Housing and urban Research, Uppsala University, Box 514, SE-75120 Uppsala, Sweden; 3 Department of Family Medicine, University of Michigan Medical School, Ann Arbor, MI USA

**Keywords:** Psychology and behaviour, Epidemiology

## Abstract

Spending time in natural environments can benefit health and well-being, but exposure-response relationships are under-researched. We examined associations between recreational nature contact in the last seven days and self-reported health and well-being. Participants (n = 19,806) were drawn from the Monitor of Engagement with the Natural Environment Survey (2014/15–2015/16); weighted to be nationally representative. Weekly contact was categorised using 60 min blocks. Analyses controlled for residential greenspace and other neighbourhood and individual factors. Compared to no nature contact last week, the likelihood of reporting good health or high well-being became significantly greater with contact ≥120 mins (e.g. 120–179 mins: ORs [95%CIs]: Health = 1.59 [1.31–1.92]; Well-being = 1.23 [1.08–1.40]). Positive associations peaked between 200–300 mins per week with no further gain. The pattern was consistent across key groups including older adults and those with long-term health issues. It did not matter how 120 mins of contact a week was achieved (e.g. one long *vs*. several shorter visits/week). Prospective longitudinal and intervention studies are a critical next step in developing possible weekly nature exposure guidelines comparable to those for physical activity.

## Introduction

A growing body of epidemiological evidence indicates that greater exposure to, or ‘contact with’, natural environments (such as parks, woodlands and beaches) is associated with better health and well-being, at least among populations in high income, largely urbanised, societies^1^. While the quantity and quality of evidence varies across outcomes, living in greener urban areas is associated with lower probabilities of cardiovascular disease^2^, obesity^3^, diabetes^4^, asthma hospitalisation^5^, mental distress^6^, and ultimately mortality^7^, among adults; and lower risks of obesity^8^ and myopia^9^ in children. Greater quantities of neighbourhood nature are also associated with better self-reported health^10–12^, and subjective well-being^13^ in adults, and improved birth outcomes^14^, and cognitive development^15^, in children.

However, the amount of greenspace in one’s neighbourhood (e.g. percent of land cover in a 1 km radius from the home), or the distance of one’s home to the nearest publically accessible green space or park^16^ is only one way of assessing an individual’s level of nature exposure. An alternative is to measure the amount of time individuals actually spend outside in natural environments^17,18^, sometimes referred to as ‘direct’ exposure^19^. Both approaches are potentially informative. Residential proximity to nature may be related to health promoting factors such as reduced air and noise pollution (although the relationships are complex^20^); and may also provide ‘indirect’ exposure via views from the property^21^. Residential proximity is also generally positively related to ‘direct’ exposure; i.e. people in greener neighbourhoods tend to report visiting greenspace more often^22^. Yet many nature visits take place outside of the local neighbourhood^23^. Moreover, such visits may compensate for a lack of nature in the neighbourhood^24^. In other words, direct exposure, or more specifically in the current context, recreational time spent in natural environments per week, cannot accurately be inferred from neighbourhood greenspace near the home.

Using data from a representative sample of the adult population of England, we aimed to better understand the relationships between time spent in nature per week and self-reported health and subjective well-being. Our research builds directly on a small number of studies that have started to look at similar issues^17,18,25,26^, and answers the call made in several recent reviews for more work in this area^27,28^. Quantification of these ‘exposure-response’ relationships can contribute to the policy process, for example by providing evidence upon which to base recommendations regarding the amount of time required to be spent in nature per week to promote positive health and well-being outcomes. A similar process was used to support development of guidelines on the amount of recommended weekly physical activity needed for health promotion and disease prevention^29^.

The research advances previous work in three key ways. First, to date, researchers have examined direct nature exposure-response relationships using either a specific visit duration^17^, or nature visit frequency over a prolonged period^26^, or both independently^18^. By multiplying the duration of a representative visit within the last week by the number of visits taken within the last week we were able to develop the first weekly exposure metric (i.e. minutes per week) for nature exposure, similar to those used in other health promotion contexts (e.g. physical activity^29^). Second, by comparing the coefficients of other, well-established, predictors of health and well-being (e.g. area deprivation) with those for average time spent in nature per week, we were able to assess the relative strength of any exposure-response relationship. Third, previous studies were constrained in their ability to look at the generalisability of relationships across different socio-demographic groups due to relatively small, geographically constrained samples. In this study, the current, nationally representative sample enabled us to stratify, *a priori*, on socio-demographic characteristics, such as age^30^, gender^31^, ethnicity^32^ and area deprivation^33^, which appeared to moderate the nature-health association in previous studies^22^.

## Results

### Models using duration categories

Descriptive data on the relationships between time spent in nature in the last 7 days (in 60 min categories) and self-reported health (Good *vs*. poor) and subjective well-being (High *vs*. low) are presented in Table 1. Percentages per category are presented for both the estimation sample (n = 19,806), and for the sample weighted to be representative of the adult population of England. Similar details for all covariates can be found in Appendix B, and relationships between our key predictor, time in nature, and all other covariates in Appendix C.

**Table 1:** The frequency and percent of respondents in each category of each predictor who reported good/very good health and high well-being.

	Self-reported health							Subjective well-being (Life satisfaction)						
	Raw Ns and %s				(Weighted %s)			Raw Ns and %s				(Weighted %s)		
	Not good		Good		Total N	Not good	Good	Low		High		Total N	Low	High
	N	%	N	%		%	%	N	%	N	%		%	%
**Nature visit exposure**														
Weekly visit duration														
≥300 mins	700	20.1	2784	79.9	3484	(18.1	81.9)	1228	35.2	2256	64.8	3484	(34.5	65.5)
240–299 mins	159	18.0	723	82.0	882	(15.5	84.5)	309	35.0	537	65.0	882	(34.1	65.9)
180–239 mins	207	20.4	807	79.6	1014	(18.1	81.9)	374	36.9	640	63.1	1014	(36.0	64.0)
120–179 mins	232	18.0	1058	82.0	1290	(15.5	84.5)	465	36.0	825	64.0	1290	(35.3	64.7)
60–119 mins	253	22.7	860	77.3	1113	(19.7	80.3)	439	39.4	674	60.6	1113	(38.2	61.8)
1–59 mins	97	27.3	258	72.7	355	(25.2	74.8)	155	43.7	200	56.3	355	(41.7	58.3)
0 mins	3678	31.5	7990	68.5	11668	(27.7	72.3)	5173	44.3	6495	55.7	11668	(42.8	57.2)
Totals	5326	*26*.*9*	14480	*73*.*1*	19806	*(23*.*5*	*76*.*5)*	8143	*41*.*1*	11663	*58*.*9*	19806	*(39*.*8*	*60*.*2)*

Notes. Weighted %s (in brackets) take into account sample weights. Similar details for all covariates available in Supplementary Table 1.

**Figure 1:**
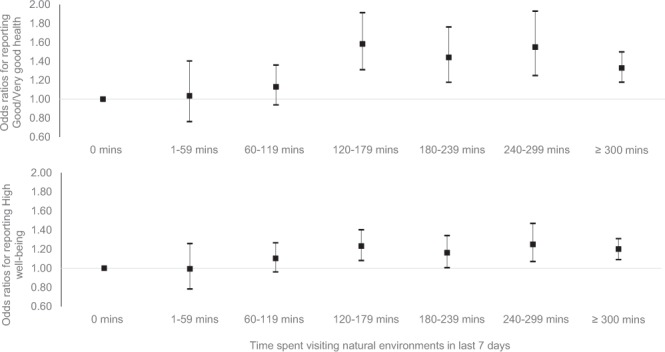
The odds ratios (OR) and 95% confidence intervals of reporting good health and high well-being as a function of nature visit duration in the last 7 days (0 mins = reference category). Note: Adjusted for urbanicity, neighbourhood greenspace, area deprivation, background PM10, sex, age, SES, restricted functioning, physical activity, employment status, relationship status, ethnicity, children in household, dog ownership and year.

The odds ratios (ORs) and 95% confidence intervals (CIs) for the survey weighted binomial logistic regressions predicting health and well-being are presented in Table 2 (full models in Appendix D). In the unadjusted models the odds ratios for reporting ‘good’ health and ‘high’ well-being were significantly higher for all nature contact ≥60 mins per week compared to 0 mins. Contact of 1–59 mins per week was not associated with better outcomes than 0 mins, and there was also no linear increase above 60 mins; longer durations were not associated with better outcomes. In the adjusted models, significance only emerged at the ≥120 mins per week category; and again additional duration was not associated with improved outcomes. The relationship appeared somewhat stronger for health than well-being (Fig. 1).

**Table 2:** The odds ratios (OR) and 95% confidence intervals (CIs) of reporting good health and high well-being as a function of nature visit duration in the last 7 days.

	Self-reported health (Good vs. poor)						Subjective well-being (High vs. low)					
	Unadjusted			Adjusted^a^			Unadjusted			Adjusted^a^		
	OR	95% CIs		OR	95% CIs		OR	95% CIs		OR	95% CIs	
		Low	High		Low	High		Low	High		Low	High
**Nature visit exposure**												
*Weekly visit duration*												
≥300 mins	1.73***	1.57	1.91	1.33***	1.18	1.50	1.42***	1.31	1.54	1.20***	1.09	1.31
240–299 mins	2.10***	1.74	2.53	1.55***	1.25	1.93	1.45***	1.24	1.68	1.25**	1.07	1.46
180–239 mins	1.74***	1.47	2.06	1.44***	1.18	1.76	1.33***	1.16	1.53	1.16*	1.00	1.34
120–179 mins	2.09***	1.79	2.44	1.59***	1.31	1.92	1.37***	1.21	1.55	1.23**	1.08	1.40
60–119 mins	1.56***	1.34	1.83	1.13	0.94	1.37	1.21**	1.06	1.39	1.10	0.96	1.27
1–59 mins	1.14	0.88	1.46	1.04	0.76	1.41	1.05	0.83	1.31	0.99	0.78	1.26
0 mins	ref	ref	ref	ref	ref	ref	ref	ref	ref	ref	ref	ref
*Covariates*												
Area	NO			YES			NO			YES		
Individual	NO			YES			NO			YES		
Constant	2.61	2.50	2.72	0.28	0.24	0.33	1.34	1.29	1.39	0.36	0.31	0.41
Pseudo R^2^	0.01			0.23			0.01			0.05		
Valid N	20,264			19,806			20,264			19,806		

Notes. ^a^Full models including covariates (urbanicity, neighbourhood greenspace, area deprivation, background PM_10_, sex, age, SES, restricted functioning, physical activity, employment status, relationship status, ethnicity, children in household, dog ownership and year) available in Supplementary Table 2. *p < 0.05; **p < 0.01; ***p < 0.001.

### Sensitivity analysis

**Figure 2:**
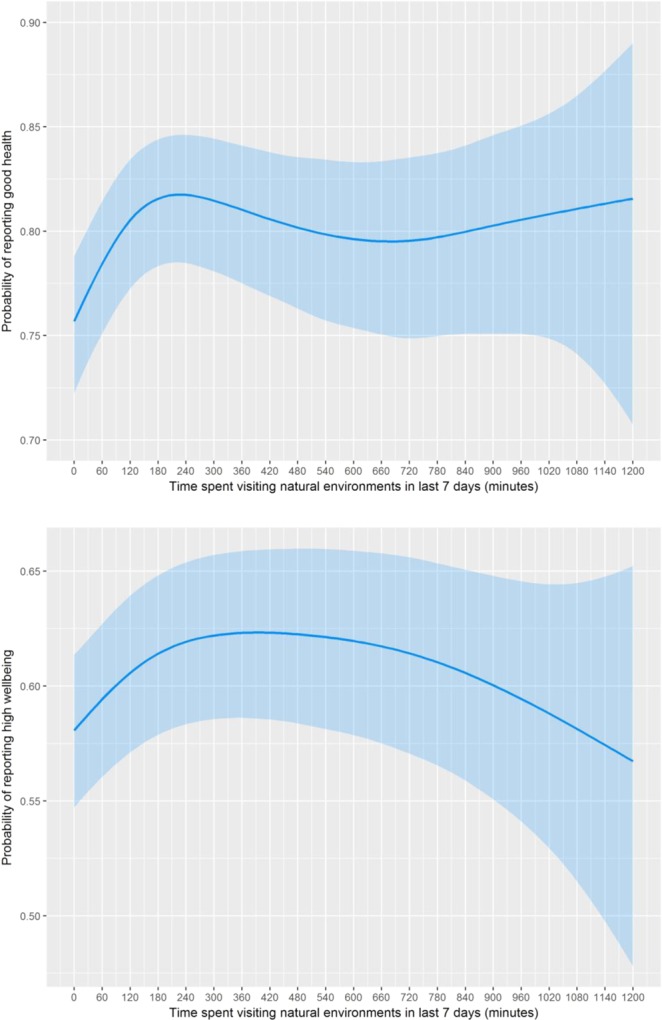
The probability of reporting (**a**) good health and (**b**) high well-being (with 95% confidence intervals) as a function of time spent in nature in the last 7 days using a generalised additive model (GAM) with a penalized cubic spline for nature contact. Note. The GAM is adjusted for urbanicity, neighbourhood greenspace, area deprivation, background PM10, sex, age, SES, restricted functioning, physical activity, employment status, relationship status, ethnicity, children in household, dog ownership and year.

We conducted three types of sensitivity analysis. First we explored exposure-response relationships using time spent in nature as a continuous variable, and outcomes modelled as binary variables using splines (Fig. 2). The figures suggested relatively steady increases in the positive relationships for both health and well-being up to around 120 mins, diminishing marginal returns from then until around 200 mins per week for health and 300 mins for well-being, and then a flattening out or even decrease thereon (though note the very large CIs > 400 mins). Although Fig. 2 should be treated with caution, due to hourly clustering (see Methods, and Appendix A, Figure C), results broadly support the categorical analyses, with some suggestion that nature exposure beyond 120 mins a week may have some additional benefits that did not emerge when health and wellbeing were treated as binary variables.

Second, we explored exposure-response relationships using time spent in nature as a categorical variable and health and wellbeing modelled as ordinal variables. Results were again very similar (Appendix E). The only slight change was significance at the 60–119 min category for both outcomes, but this finding is not easily comparable to the binary logistic results for reasons explained in more detail in Appendix E.

Our final sensitivity analysis modelled both time and well-being as continuous variables (Appendix E, Figure D). Again the results were very similar to the original model (Fig. 2b). Due to the inherently ordinal structure of the general health variable, we were unable to conduct a comparable sensitivity model for health.

### Contextualisation of results

**Figure 3:**
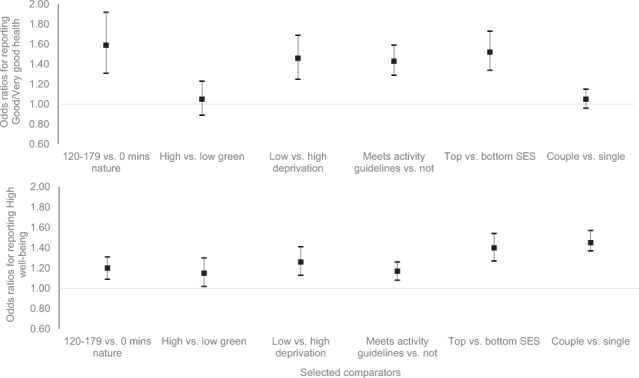
The odds ratios (OR) and 95% confidence intervals of reporting good health and high well-being as a function of nature visits and selected covariates (controlling for all other covariates).

To contextualise the magnitude of the relationship between weekly nature contact and health and well-being, Fig. 3 presents the relevant ORs (CIs) alongside those for selected predictors including: neighbourhood greenspace and deprivation; physical exercise; individual SES; and relationship status (see Appendix D for details on all covariates). The figure highlights that 120–179 mins *vs*. 0 mins of nature contact per week was associated with: (a) a similar likelihood of reporting good health as, living in an area of low *vs*. high deprivation; meeting *vs*. not meeting physical activity guidelines, and (c) being in a high *vs*. low SES occupation. Although the association between nature contact at this level and wellbeing was similar to that between high *vs*. low: greenspace, deprivation and physical activity; it was less important than SES and relationship status.

### Generalisability of results

Table 3 shows results of analyses stratified on key area and individual level factors (see Appendix F for full details). For these analyses, nature contact was reconfigured into three duration levels reflecting: (a) ‘no exposure’ (0 minutes, ref); (b) ‘low exposure’, not associated with significantly greater likelihood of good health and high wellbeing (1–119 mins); and (c) ‘high exposure’, i.e. all durations associated with significantly higher likelihood of good health and high well-being combined (≥120 mins). Estimates from the models of health showed that the positive relationship found for ‘high’ but not ‘low’ exposure, compared to ‘no exposure’, in the overall model was consistent across those living in urban and rural, and high and low deprivation, areas. It was also consistent for: both males/females; those above/below 65years old; those of high/low occupational social grade; those with/without a long-term illness/disability; and for those who did *vs*. did not meet physical activity recommendations. Stratification on neighbourhood greenspace suggested those in areas of high (but not low) greenspace also had greater odds of good health if they spent any time in nature per week compared to 0 mins, possibly reflecting the importance of indirect exposure among this cohort. Stratification on ethnicity showed the threshold was maintained amongst white British, but not ‘other’ respondents. Stratified models of well-being showed that ‘high’ but not ‘low’ exposure was associated with significantly greater odds of high wellbeing in all cases.

**Table 3:** The odds ratios (OR) and 95% confidence intervals (CIs) of reporting good health and high well-being as a function of the three main categories of nature visit duration in the last 7 days, stratified on key area and individual covariates.

	Self-reported health (Good vs. poor)						Subjective well-being (High vs. low)					
	1–119 mins			≥120 mins			1–119 mins			≥120 mins		
	OR	95% CIs		OR	95% CIs		OR	95% CIs		OR	95% CIs	
		Low	High		Low	High		Low	High		Low	High
**Area level stratification**												
*Urbanicity*												
Urban (n = 18,694)	1.08	0.91	1.27	1.38***	1.25	1.52	1.07	0.94	1.21	1.19***	1.11	1.28
Rural (n = 1,112)	1.94	0.96	3.95	2.27***	1.49	3.47	1.37	0.79	2.39	1.59**	1.17	2.18
*Area greenspace*												
High (n = 8,510)	1.48**	1.16	1.90	1.54***	1.34	1.78	1.05	0.87	1.27	1.28***	1.14	1.42
Low (n = 11,966)	0.88	0.71	1.09	1.33***	1.17	1.52	1.11	0.94	1.30	1.16**	1.06	1.27
*Area deprivation*												
High (n = 11,796)	1.03	0.84	1.27	1.36***	1.20	1.54	1.10	0.94	1.29	1.29**	1.17	1.41
Low (n = 8,010)	1.23	0.95	1.59	1.53***	1.32	1.78	1.13	0.93	1.36	1.23**	1.10	1.37
**Individual level stratification**												
*Sex*												
Female (n = 10,419)	0.99	0.79	1.23	1.38***	1.21	1.58	1.03	0.88	1.22	1.22***	1.11	1.35
Male (n = 9,387)	1.25	0.98	1.58	1.46***	1.27	1.67	1.12	0.94	1.34	1.20**	1.08	1.32
*Age*												
16–64 yrs (n = 14,667)	1.05	0.87	1.27	1.28***	1.14	1.44	1.07	0.94	1.29	1.18***	1.09	1.28
65+yrs (n = 5,193)	1.27	0.91	1.76	1.87***	1.57	2.22	1.10	0.84	1.43	1.35***	1.16	1.57
*Restricted functioning*												
Yes (n = 4,545)	1.31	0.97	1.77	1.42***	1.20	1.68	1.07	0.80	1.43	1.31***	1.12	1.53
No (n = 15,261)	1.05	0.87	1.26	1.42***	1.27	1.60	1.08	0.94	1.23	1.19***	1.10	1.29
*SES*												
AB/C1 (highest) (n = 8,624)	1.09	0.86	1.39	1.43***	1.24	1.65	1.01	0.85	1.20	1.16**	1.05	1.28
C2/DE (lowest) (n = 11,182)	1.16	0.94	1.44	1.43***	1.26	1.62	1.18	1.00	1.40	1.29**	1.17	1.43
*Ethnicity*												
White British (n = 15,198)	1.14	0.96	1.36	1.47***	1.33	1.63	1.12	0.97	1.29	1.21***	1.11	1.31
Other (n = 4,608)	1.01	0.69	1.48	1.21	0.95	1.53	0.96	0.75	1.21	1.22*	1.05	1.42
*Meets activity guidelines*												
No (n = 15,008)	1.13	0.95	1.36	1.48***	1.32	1.65	1.05	0.92	1.20	1.17***	1.08	1.27
Yes (n = 4,798)	0.98	0.67	1.43	1.22*	1.01	1.47	1.21	0.92	1.60	1.32***	1.14	1.51

Notes. In all analyses the reference duration is 0 minutes per week; All analyses control for area and individual covariates not being stratified. Full models available on request. *p < 0.05; **p < 0.01; ***p < 0.001.

Additional analyses found no differences in health and well-being as a function of how ‘high’ exposure was achieved (a) one 120+ min visit; (b) two 60+ min visits; or (c) or three/more ≤ 40 min visits (see Appendix G for details).

## Discussion

Growing evidence of a positive association between contact with natural environments and health and well-being has led to calls for improved understanding of any exposure-response relationships^27,28^. The aim of the current study was to assess these relationships with a measure based on direct exposure to natural environments, rather than residential proximity, using data from a large nationally representative sample in England. Exposure was defined in terms of the self-reported minutes spent in natural environments for recreation in the last seven days; and outcomes were self-reported health and subjective well-being.

After a range of covariates had been taken into account, individuals who spent between 1 and 119 mins in nature in the last week were no more likely to report good health or high well-being than those who reported 0 mins. However, individuals who reported spending ≥120 mins in nature last week had consistently higher levels of both health and well-being than those who reported no exposure. Sensitivity analyses using splines to allow duration to be modelled as a continuous variable suggested that beyond 120 mins there were decreasing marginal returns until around 200–300 mins when the relationship flattened or even dropped. We tentatively suggest, therefore, that 120 mins contact with nature per week may reflect a kind of “threshold”, below which there is insufficient contact to produce significant benefits to health and well-being, but above which such benefits become manifest.

In terms of magnitude, the association between health, well-being and ≥120 mins spent in nature a week, was similar to associations between health, well-being and: (a) living in an area of low *vs*. high deprivation; (b) being employed in a high *vs*. low social grade occupation; and (c) achieving *vs*. not achieving recommended levels of physical activity in the last week. Given the widely stated importance of all these factors for health and well-being, we interpret the size of the nature relationship to be meaningful in terms of potential public health implications.

That the ≥120 mins “threshold” was present even for those who lived in low greenspace areas reflects the importance of measuring recreational nature contact directly when possible, rather than simply using residential proximity as a proxy for all types of nature exposure. People travel beyond their local neighbourhoods to access recreational nature experiences, and indeed in our own data those who lived in the least green areas had higher odds of spending ≥120 mins in nature than those living in greener neighbourhoods (Appendix C). Impoverished local opportunities need not be a barrier to nature exposure^23,24^. That the “threshold” was also present for those with long-term illnesses/disability, suggests that the positive overall association in the data was not simply due to healthier people visiting nature more often.

One explanation for our findings might be that time spent in nature is a proxy for physical activity, and it is this which is driving the relationship, not nature contact *per se*. In England, for instance, over 3 million adults achieve recommended activity levels fully, or in part, in natural settings^34^. Although: (a) we tried to control for this by including physical activity over the last 7 days in our models; and (b) the threshold applied to individuals who did not meet activity guidelines; we were unable to fully untangle these issues. Experimental research, however, indicates that some benefits cannot be due solely to physical activity. Research into shinrin-yoku (Japanese “forest bathing”)^35^, for instance, suggested that various psycho-physiological benefits can be gained from merely sitting passively in natural *vs*. urban settings. Moreover, physical activity conducted in nature may be more psychologically beneficial than in other locations^36^, suggesting a complex interaction between the two which requires further research to fully understand^20^.

The current results also suggested that it did not matter how the “threshold” was achieved. This may be because individuals selected exposures to fit their personal preferences and circumstances. For instance, some may prefer long walks on the weekend in locations further from home; while others may prefer regular shorter visits to parks in the local area. To recommend the former type of person stops their long weekly visit in favour of several shorter trips or *vice versa* may be misguided.

Whilst this study deepens our understanding of the potential value of spending time outdoors in nature to health and well-being, it is too early to make specific guidance due to several limitations. First, the data are observational and cross-sectional; and thus, notwithstanding the same pattern holding for those with a long-term illness/disability, we are unable to rule out the possibility that the association is, at least in part, due to healthier, happier people spending more time in nature. Prospective longitudinal studies of the kind used to help develop physical activity guidelines^29^, and nature-based intervention studies are needed to better understand causality. Cimprich and Ronis^37^, for instance, found that women recently diagnosed with breast cancer scored higher on several attention tasks, compared to standard care controls, following a five-week period of spending *120 mins per week* in ‘natural restorative environments’. The authors argued that the 120 mins per week of nature exposure helped the women restore cognitive resources depleted by the stress of their diagnoses and early treatment. Although our sample was more heterogeneous, weekly nature exposure may work in a similar fashion by reducing generally high levels of stress^38^. Similar studies are needed to see how generalizable any potential “threshold” is across a range of situations, and to see how long an individual needs to maintain a certain amount of weekly exposure to achieve health and well-being gains. Although effects on attentional processes were observed after just 5 weeks in Cimprich and Ronis^37^, health effects may need longer; and it is also important to see whether different types of nature contact might confer different benefits.

We also note that, although significant, time in nature explained relatively little variance in either health or wellbeing in these models based on cross-sectional data (approx. 1% in unadjusted models in both cases). It will therefore be important to explore effect sizes in prospective/experimental studies to better understand the cost/benefit implications of any potentials interventions.

Another limitation concerned our estimate of weekly exposure. As duration was asked about only a single randomly selected visit in the last week, we assumed that at the population level this was representative of all visits. Although rigorous collection protocols meant that the effects of a typical visit selection are likely to cancel out over a sample of nearly 20,000, we recognise that accuracy at the individual level would be improved if duration were asked about all visits in the last week. We also acknowledge that our data rely on self-reports and thus results needed to be treated with caution. For instance, self-reported duration is likely to be less accurate than measures obtained from geo-tracking individuals during specific visits^39^, or over several days^40^, and individuals may have been unsure about, or reluctant to discuss, certain issues which were included as covariates (e.g. long standing illness/disability). Future studies would ideally collate as much data via non self-report measures as possible. We note, moreover, that unlike exposure to often invisible environmental factors such as air pollution, we can potentially ‘re-live’ our experiences of the natural world in memory, for instance during periods of ‘mind wandering’, and derive benefits from these recollections independent of those experienced *in situ*^41^. Thus, an exposure in this context may be considered as the time *in situ* plus all subsequent time spent thinking about the experience^42^. In short, we believe further work is needed to think more critically and creatively about what the term ‘exposure’ means in the current context.

We also remain cautious about any potential ≥120 mins “threshold”. In part its emergence may be a consequence of the clustering of duration responses around the hour mark and subsequent stratification, rather than anything materially different occurring at this level of exposure. The spline models, for instance, suggested a more nuanced pattern. However, this smoothing of the data was still reliant on a highly non-normal distribution, suggesting that we need to be cautious about these analyses as well. Further work is also needed to explore the ‘peak’ of returns at around 200–300 mins, to better understand why spending more time in nature is associated with little marginal gain. Thus, we see the tentative “threshold” and “peak” discussed here more as a starting points for discussion and further investigation, than clearly established findings.

Finally, our results say little about exposure ‘quality’. Research considering the quality of the natural environment in terms of plant and/or animal species richness suggests that experiences may be better in more biodiverse settings^25,43^. Contact with nature is more than just a complex multi-sensory experience, to varying degrees personal histories and meanings, longstanding cultural practices, and a sense of place play some role in the benefits realised^44–46^, factors which may account for why we did not find the same pattern for health individuals not identifying as White British. In the current research, for instance, exposure estimates relied upon visits undertaken voluntarily, presumably because they had features important to those individuals^47^ and these effects may not be found if individuals were to regularly spend 120 mins a week in a natural environment of less personal relevance (e.g. those who self-identified as ‘White European’). Our estimates also explicitly excluded time spent in one’s own garden which can be an important form of meaningful nature contact for many people^48^. All of these issues will need greater consideration in future research.

To conclude, although this research suggests that spending ≥120 mins a week in nature may be an important “threshold” for health and well-being across a broad range of the adult population in England, we believe that more prospective cohort, longitudinal, and experimental studies are required before any clear conclusions can be drawn. In addition to improving the duration-exposure estimates used here, more research is also needed to understand the impact of different activities undertaken, as well as the effect of environmental quality and personal meaning. Nevertheless, we see our findings as an important starting point for discussions around providing simple, evidence-based recommendations about the amount of time spent in natural settings that could result in meaningful promotion of health and well-being.

## Methods

### Participants & procedure

Participants were drawn from Waves 6 and 7 (2014–2015/2015–2016) of the Monitor of Engagement with the Natural Environment (MENE) survey (the only Waves where our key outcomes were consistently measured). The survey, which is part of the UK government’s National Statistics, is repeat cross-sectional (different people take part in each wave), and is conducted across the whole of England and throughout the year (approx. 4,000 people per week) to reduce potential geographical and seasonal biases^49^. As part of the UK’s official statistics, sampling protocols are extensive, to ensure as representative a sample of the adult English population as possible. Full details can be found in the annual MENE Technical Reports^49^ with key features including: (a) “a computerised sampling system which integrates the Post Office Address file with the 2001 Census small area data at output area level. This enables replicated waves of multi-stage stratified samples”; (b) “the areas within each Standard Region are stratified into population density bands and within band, in descending order by percentage of the population in socio-economic Grade I and II”; (c) “[in order to] maximise the statistical accuracy of the sampling, sequential waves of fieldwork are allocated systematically across the sampling frame to ensure maximum geographical dispersion”; (d) “to ensure a balanced sample of adults within the effective contacted addresses, a quota is set by sex (male, female housewife, female non-housewife); within the female housewife quota, presence of children and working status and within the male quota, working status”; and (e) “the survey data is weighted to ensure that the sample is representative of the UK population in terms of the standard demographic characteristics” (ref.^49^, p.5). Data is collected using in-home face-to-face interviews with responses recorded using Computer Assisted Personal Interviewing (CAPI) software.

Although the total sample for these years was n = 91,190, the health and well-being questions were only asked in every fourth sampling week (i.e. monthly, rather than weekly) resulting in a reduced sample of n = 20,264. In order to account for any residual biases in sampling at this monthly level, special ‘month’ survey weights are included in the data set. These were applied in the current analysis to ensure that results remained generalisable to the entire adult population of England. All data were anonymised by Natural England and are publically accessible at: http://publications.naturalengland.org.uk/publication/2248731?category=47018. Ethical approval was not required for this secondary analysis of publically available National Statistics.

### Outcomes: Self-reported health & subjective well-being

Self-reported health (henceforth: *health*) was assessed using the single-item: ‘How is your health in general?’ (sometimes referred to as ‘SF1’). Response options were: ‘Very bad’, ‘Bad’, ‘Fair’, ‘Good’ and ‘Very good’. Responses are robustly associated with use of medical services^50^ and mortality^51^; and crucially, for current purposes, neighbourhood greenspace^13^. Following earlier work we dichotomised responses into ‘Good’ (‘Good/very good’, weighted = 76.5%) and ‘Not good’ (‘Fair/bad/very bad’, 23.5%)^52^. Subjective well-being (henceforth: *well-being*) was assessed using the ‘Life Satisfaction’ measure, one of the UK’s national well-being measures^53^: ‘*Overall how satisfied are you with life nowadays?’* with responses ranging from 0 ‘Not at all’ to 10 ‘Completely’. Again, following earlier studies we dichotomised responses into ‘High’ (8–10, 60.2%) and Low (0–7, 39.8%) well-being^54^. Histograms of the (non-normal) distributions for both outcome variables are presented in Appendix A. Of note although the dichotomisation points were based on prior research, they are consistent with the current data; the 50^th^ percentile for health was in the ‘good’ response and for wellbeing in ‘8’. Sensitivity analyses conducted on ordinal (both health and wellbeing) and linear (wellbeing only) variations of these variables are presented in Appendix E.

### Exposure: Recreational nature contact in last 7 days

Recreational nature contact, or time spent in natural environments in the last week, was derived by multiplying the number of reported recreational visits per week by the length of a randomly selected visit in the last week. Participants were introduced to the survey as follows: “*I am going to ask you about occasions in the last week when you spent your time out of doors*. *By out of doors we mean open spaces in and around towns and cities*, *including parks*, *canals and nature areas; the coast and beaches; and the countryside including farmland*, *woodland*, *hills and rivers*. *This could be anything from a few minutes to all day*. *It may include time spent close to your home or workplace*, *further afield or while on holiday in England*. *However this does not include: routine shopping trips or; time spent in your own garden*.” Then they were asked “*how many times*, *if at all*, *did you make this type of visit yesterday/on* <*DAY*> ” for each of the previous seven days. Ninety-eight percent of respondents reported ≤7 visits last week. The remaining 2% were capped at 7 visits to avoid dramatically skewing weekly duration estimates.

After basic details of each visit (up to 3 per day) were recorded, a single visit was selected at random by the CAPI software, for the interviewer to ask further questions about, including: *“How long did this visit last altogether?” (Hours & Minutes)*. Due to random selection, even if the selected visit was not necessarily representative for any given individual, the randomisation procedure should reduce potential bias at the population level at which our analyses were conducted. Weekly duration estimates were thus derived by multiplying the duration for this randomly selected visit by the number of stated visits in the last seven days (capped at 7). Following the approach of earlier exposure-response studies in the field (e.g. Shanahan *et al*., 2016), duration was categorised into 7 categories: 0 mins (n = 11,668); 1–59 mins (n = 355); 60–119 mins (n = 1,113); 120–179 mins (n = 1,290); 180–239 mins (n = 1,014); 240–299 mins (n = 882); ≥300 mins (n = 3,484). An alternative banding at 30 minutes was problematic because of very low Ns for some bands (e.g. 1–29 mins, n = 85), reflecting the fact that weekly duration estimates clustered around the hour marks, e.g. 78% of the unweighted observations within the 120–179 mins band were precisely 120 mins (See Appendix A, Figure C for duration histogram). The highest band was capped at ≥300 mins due to the large positive skew of the data.

### Control variables

Health and well-being are associated with socio-demographic and environmental characteristics at both neighbourhood (e.g. area deprivation) and individual (e.g. relationship status) levels^55^. As many of these variables may also be related to nature exposure they were controlled for in the adjusted analyses.

#### Area level control variables

Area level covariate data was assigned on the spatial level of the Census 2001 Lower-layer Super Output Areas (LSOAs) in which individuals lived. There were 32,482 LSOAs in England, each containing approximately 1,500 people within a mean physical area of 4km^2^.

### Neighbourhood greenspace

In order to understand how much greenspace is in an individual’s neighbourhood, we derived an area density metric using the Generalised Land Use Database (GLUD)^56^. The GLUD provides, for each LSOA in England, the area covered by greenspace and domestic gardens. These were summed and divided by the total LSOA area to provide the greenspace density metric. This metric was allocated to each individual in the sample, based on LSOA of residence. Following previous literature, individuals were assigned to one of five quintiles of greenspace based on this definition (ranging from least green to most green)^33^. Rather than derive quintiles of greenspace from the current sample (i.e. divide the current sample into five equal parts based on the percentage of greenspace in their LSOA), we assigned individuals instead to one of five pre-determined greenspace quintiles based on the distribution of greenspace across all 32,482 LSOAs in England. Although this meant that we did not get exactly equal 20% shares of our current sample across greenspace quintiles (although due to the sampling protocol we were still very close to this, see Appendix B) this approach allowed inferences to be made across the entire country, rather than simply to the current sample. In exploratory sensitivity analyses we defined greenspace as the GLUD category ‘greenspace’ only, with the GLUD category ‘gardens’ excluded. This produced very similar results, so we focused on the more inclusive definition including both aspects. In further exploratory sensitivity analyses, we assigned individuals to five greenspace categories defined by equal ranges of greenspace coverage (e.g. 0–20%, 21–40%, 41–60% etc.) rather than quintiles based on percentages of the population. This also produced very similar results, so again we decided to go with the most common approach. In subsequent analyses the least green quintile acted as the reference category.

### Area deprivation

Each LSOA in England is assessed in terms of several parameters of deprivation, including unemployment and crime, levels of educational, income, health metrics, barriers to housing and services, and the living environment. A total Index of Multiple Deprivation (IMD) score is derived from these subdomains^57^. Following previous studies^52^, we assigned individuals into deprivation quintiles based on the LSOA in which they lived. As with greenspace, the cut points for area deprivation quintiles were also based on all LSOAs in England, rather than those in the current sample, to allow inference to the population as a whole (most deprived quintile = *ref*).

### Air pollution

An indicative measure of air pollution was operationalised as LSOA background PM_10_ assigned to tertiles of all LSOAs in England (lowest particulate concentration = *ref*). PM10 concentrations, based on Pollution Climate Mapping (PCM) model simulations^58^, were averaged over the period 2002–2012, and aggregated from 1 km square resolution to LSOAs.

#### Individual level controls

Individual level controls comparable to earlier studies in this area^6,7,12,13,15^ included: sex (male = *ref*); age (categorised as 16–64 = *ref*; 65+); occupational social grade (AB (highest, e.g. managerial), C1, C2 and DE (lowest, e.g. unskilled labour, = *ref*) as a proxy for individual socio-economic status (SES); employment status (full-time, part-time, in education, retired, not working/unemployed = *ref*); relationship status (married/cohabiting; single/separated/divorced/widowed = *ref*); ethnicity (White British; other = *ref*); number of children in the household (≥1 *vs*. 0 = *ref*); and dog ownership (Yes; No = *ref*).

Two further control variables were particularly important. First, the survey asked: ‘*Do you have any long standing illness*, *health problem or disability that limits your daily activities or the kind of work you can do?*’ (‘Restricted functioning’: Yes; No = *ref*). Including this variable, at least in part, controls for reverse causality. If similar associations between nature exposure and health and well-being are found for both those with and without restricted functioning, this would support the notion that the associations are not merely due to healthier, more mobile people visiting nature more often.

We also controlled for the number of days per week people reported engaging in physical activity >30 mins; in the current analysis dichotomised as either meeting or not meeting guidelines of 150 mins per week (i.e. 5 days in the week with physical activity >30 mins). Some people achieve this guideline though physical activity in natural settings^35^, thus, any association between time spent in nature and health may simply be due to the physical activity engaged in these settings. We believe this is not the case in the current context because the (rank order) correlation between weekly nature contact and the number of days a week an individual engaged in >30 mins of physical activity was just r_s_ = 0.27. Nevertheless, by controlling for weekly activity levels, modelled relationships between time in nature and health have less bias from this source, and, therefore, improved estimates of association with nature exposure *per se*.

#### Temporal controls

Due to the multi-year pooled nature of the data, year/wave was also controlled for. Preliminary analysis found no effect of the season in which the data were collected so this was excluded from final analyses.

### Analysis strategy

Survey weighted binomial logistic regressions were used to predict the relative odds that an individual would have ‘Good’ health or ‘High’ well-being as a function of weekly nature exposure in terms of duration categories per week. Model fit was provided by pseudo R^2^; here the more conservative Cox and Snell estimate. The outcome binary variables were first regressed against the exposure duration categories to test direct relationships; adjusted models were then specified to include the individual and area level control variables. Due to missing area level data for a small minority of participants (n = 456), our estimation samples for these adjusted models were n = 19,808. Preliminary analysis found that the weighted descriptive proportions among this reduced estimation sample differed only negligibly from those among all available observations in the wider MENE sample, suggesting our complete case analysis approach did not distort the population representativeness of the estimation sample. The full n = 20,264 sample was maintained for the unadjusted model to provide the most accurate, weighted representation of the data, as reducing unadjusted models to n = 19,808 produced practically identical results. Although our main analyses used duration *categories* of weekly nature contact, an exploratory analysis used generalized additive models incorporating a penalized cubic regression spline of duration as a continuous variable (adjusting for the same set of covariates). This enabled us to produce a ‘smoother’ plot of the data. Analyses and plotting was done using R version 3.4.1, using packages mgcv and visreg^59^.

To explore the generalisability of any pattern across different socio-demographic groups, we also *a priori* stratified the analyses on several area and individual covariates (as defined above) which have been found to be important in previous studies: (a) Urbanicity; (b) Neighbourhood greenspace; (c) Area deprivation; (d) Sex; (e) Age; (f) Restricted functioning; (g) Individual socio-economic status (SES); (f) Ethnicity; and (g) Physical activity. In the case of the three multi-category predictors (area greenspace/deprivation, individual SES), binary classifications were derived for the stratified analyses to maintain robust sample sizes in each category. In the case of LSOA greenspace and deprivation binary splits were made based on the median cut-point for all LSOAs in England; SES was dichotomised by collapsing the social grade categories in the standard way, A/B/C1 *vs*. C2/D/E.

## Supplementary information

Supplementary Materials
