# Supplementary material for: Spending at least 120 minutes a week in nature is associated with good health and wellbeing
Source: Sci Rep. 2019 Jun 13;9:7730. doi: 10.1038/s41598-019-44097-3 (PMC6565732; doi:10.1038/s41598-019-44097-3)
Supplement: Supplementary file 1 — Supplementary Materials [file 41598_2019_44097_MOESM1_ESM.pdf]

Spending at least 120 minutes a week in nature is associated with good health and wellbeing

Mathew White, Ian Alcock, James Grellier, Benedict Wheeler, Terry Hartig, Sara Warber, Angie Bone, Michael Depledge & Lora Fleming

Appendix A: Response distributions of the health and wellbeing outcomes

Figure A: Histogram of self-reported health.

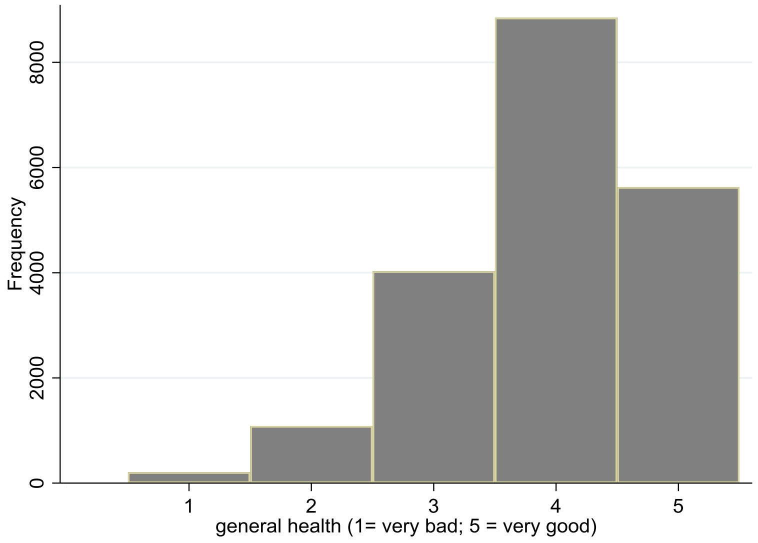

Figure B: Histogram of life satisfaction

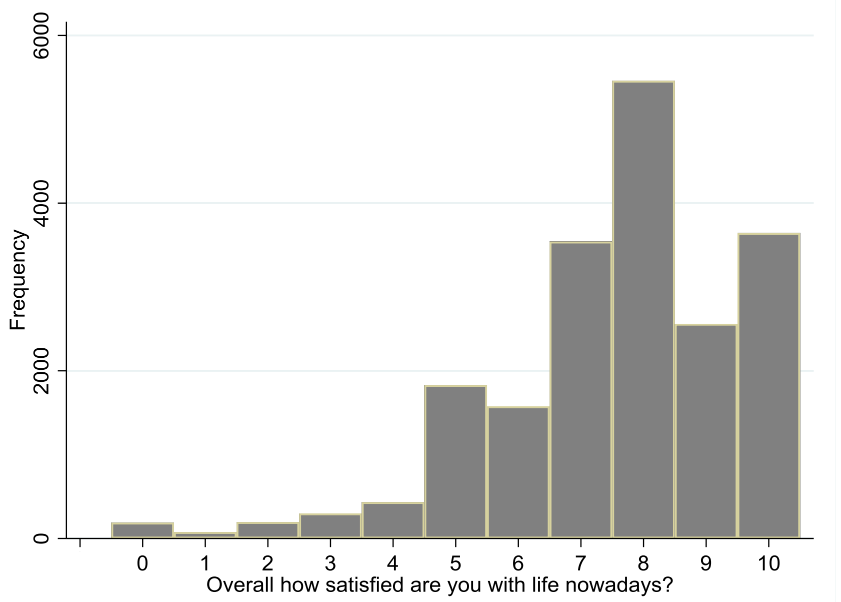

Figure C: Histogram of recreational contact with nature per week (in minutes)

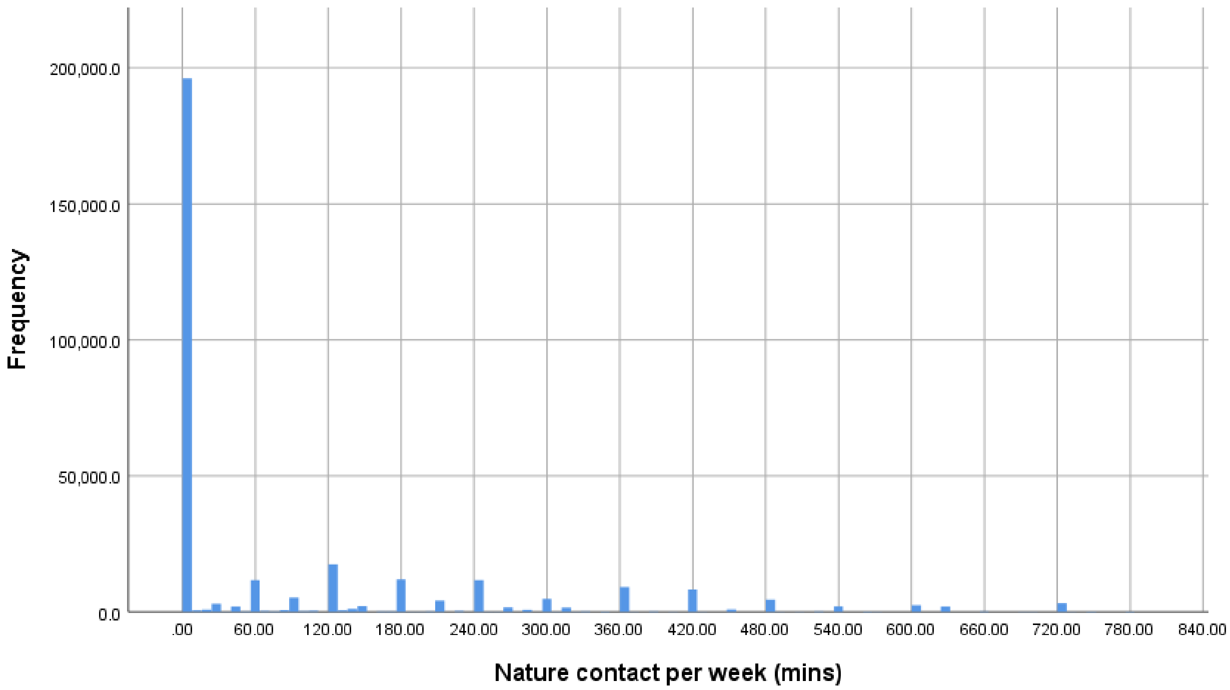

N.B. Duration capped at 800 minutes for ease of viewing. The clustering of responses around the hour marks (60, 120, 180, 240, 300, 360, 420 minutes etc.) is clearly visible.

Appendix B: The frequency and percent of respondents in each category of each predictor who reported good/very good health and high well-being.

Supplementary Table S1: The frequency and percent of respondents in each category of each predictor who reported good/very good health and high well-being.

	Self-reported health							Subjective well-being						
	Raw Ns and %s					(Weighted %s)		Raw Ns and %s					(Weighted %s)	
	Not good		Good			Not good	Good	Low		High			Low	High
	N	%	N	%	Total N	%	%	N	%	N	%	Total N	%	%
**Nature visit exposure**														
Weekly visit duration														
≥300 mins	700	20.1	2784	79.9	*3484*	(18.1	81.9)	1228	35.2	2256	64.8	*3484*	(34.5	65.5)
240-299 mins	159	18.0	723	82.0	*882*	(15.5	84.5)	309	35.0	537	65.0	*882*	(34.1	65.9)
180-239 mins	207	20.4	807	79.6	*1014*	(18.1	81.9)	374	36.9	640	63.1	*1014*	(36.0	64.0)
120-179 mins	232	18.0	1058	82.0	*1290*	(15.5	84.5)	465	36.0	825	64.0	*1290*	(35.3	64.7)
60-119 mins	253	22.7	860	77.3	*1113*	(19.7	80.3)	439	39.4	674	60.6	*1113*	(38.2	61.8)
1-59 mins	97	27.3	258	72.7	*355*	(25.2	74.8)	155	43.7	200	56.3	*355*	(41.7	58.3)
0 mins	3678	31.5	7990	68.5	*11668*	(27.7	72.3)	5173	44.3	6495	55.7	*11668*	(42.8	57.2)
Totals	*5326*	*26.9*	*14480*	*73.1*	*19806*	(*23.5*	*76.5*)	*8143*	*41.1*	*11663*	*58.9*	*19806*	(*39.8*	*60.2*)
**Area level covariates**														
Urbanicity														
Rural	285	25.6	827	74.4	*1112*	(22.4	77.6)	365	32.8	747	67.2	*1112*	(32.0	68.0)
Urban/town fringe	5041	27.0	13653	73.0	*18694*	(23.5	76.5)	7778	41.6	10916	58.4	*18694*	(40.2	59.8)
Totals	*5326*	*26.9*	*14480*	*73.1*	*19806*	(*23.5*	*76.5*)	*8143*	*41.1*	*11663*	*58.9*	*19806*	(*39.7*	*60.3*)
Neighbourhood green														
Greenest quintile	1054	26.6	2909	73.4	*3963*	(23.0	77.0)	1386	35.0	2577	65.0	*3963*	(33.6	66.4)
Quintile 2	1056	26.6	2910	73.4	*3966*	(23.6	76.4)	1616	40.7	2350	59.3	*3966*	(39.4	60.6)
Quintile 3	1165	29.4	2792	70.6	*3957*	(25.4	74.6)	1688	42.7	2269	57.3	*3957*	(40.5	59.5)

Quintile 4	1074	27.2	2880	72.8	*3954*	(23.7	76.3)	1662	42.0	2292	58.0	*3954*	(41.2	58.8)
Least green quintile	977	24.6	2989	75.4	*3966*	(21.6	78.4)	1791	45.2	2175	54.8	*3966*	(44.1	55.9)
Totals	*5326*	*26.9*	*14480*	*73.1*	*19806*	(*23.5*	*76.5*)	*8143*	*41.1*	*11663*	*58.9*	*19806*	(*39.7*	*60.3*)
Area Deprivation														
Least deprived quintile	855	21.2	3182	78.8	*4037*	(19.2	80.8)	1375	34.1	2662	65.9	*4037*	(33.3	66.7)
Quintile 2	894	24.1	2810	75.9	*3704*	(21.0	79.0)	1392	37.6	2312	62.4	*3704*	(36.6	63.4)
Quintile 3	1007	26.9	2730	73.1	*3737*	(24.1	75.9)	1486	39.8	2251	60.2	*3737*	(38.7	61.3)
Quintile 4	1155	28.5	2902	71.5	*4057*	(24.6	75.4)	1815	44.7	2242	55.3	*4057*	(43.6	56.4)
Most deprived quintile	1415	33.1	2856	66.9	*4271*	(29.3	70.7)	2075	48.6	2196	51.4	*4271*	(47.4	52.6)
Totals	*5326*	*26.9*	*14480*	*73.1*	*19806*	(*23.5*	*76.5*)	*8143*	*41.1*	*11663*	*58.9*	*19806*	(*39.7*	*60.3*)
Air pollution														
PM_{10} lowest tertile	1930	29.2	4685	70.8	*6615*	(25.4	74.6)	2569	38.8	4046	61.2	*6615*	(37.3	62.7)
PM_{10} middle tertile	1784	27.0	4832	73.0	*6616*	(23.5	76.5)	2682	40.5	3934	59.5	*6616*	(39.1	60.9)
PM_{10} highest tertile	1612	24.5	4963	75.5	*6575*	(21.5	78.5)	2892	44.0	3683	56.0	*6575*	(42.7	57.3)
Totals	*5326*	*26.9*	*14480*	*73.1*	*19806*	(*23.5*	*76.5*)	*8143*	*41.1*	*11663*	*58.9*	*19806*	(*39.7*	*60.3*)
**Individual covariates**														
Sex														
Male	2528	26.9	6859	73.1	*9387*	(22.8	77.2)	3955	42.1	5432	57.9	*9387*	(41.0	59.0)
Female	2798	26.9	7621	73.1	*10419*	(24.1	75.9)	4188	40.2	6231	59.8	*10419*	(38.4	61.6)
Totals	*5326*	*26.9*	*14480*	*73.1*	*19806*	(*23.5*	*76.5*)	*8143*	*41.1*	*11663*	*58.9*	*19806*	(*39.7*	*60.3*)
Age														
16-64 years	3223	22.0	11444	78.0	*14667*	(19.2	80.8)	6273	42.8	8394	57.2	*14667*	(41.0	59.0)
≥ 65 years	2103	40.9	3036	59.1	*5139*	(38.7	61.3)	1870	36.4	3269	63.6	*5139*	(35.1	64.9)
Totals	*5326*	*26.9*	*14480*	*73.1*	*19806*	(*23.5*	*76.5*)	*8143*	*41.1*	*11663*	*58.9*	*19806*	(*39.7*	*60.3*)
Socio-economic status														
AB (Highest)	637	18.3	2835	81.7	*3472*	(17.8	82.2)	1131	32.6	2341	67.4	*3472*	(32.8	67.2)

C1	1016	19.7	4136	80.3	*5152*	(19.3 | 80.7)	1962	38.1	3190	61.9	*5152*	(38.3 | 61.7)
C2	905	22.7	3088	77.3	*3993*	(21.9 | 78.1)	1599	40.0	2394	60.0	*3993*	(40.6 | 59.4)
DE (Lowest)	2768	38.5	4421	61.5	*7189*	(35.5 | 64.5)	3451	48.0	3738	52.0	*7189*	(47.5 | 52.5)
Totals	*5326*	*26.9*	*14480*	*73.1*	*19806*	(*23.5* | *76.5*)	*8143*	*41.1*	*11663*	*58.9*	*19806*	(*39.7* | *60.3*)
Restricted functioning														
No	2112	13.8	13149	86.2	*15261*	(12.6 | 87.4)	5685	37.3	9576	62.7	*15261*	(36.7 | 63.3)
Yes	3214	70.7	1331	29.3	*4545*	(68.2 | 31.8)	2458	54.1	2087	45.9	*4545*	(52.0 | 48.0)
Totals	*5326*	*26.9*	*14480*	*73.1*	*19806*	(*23.5* | *76.5*)	*8143*	*41.1*	*11663*	*58.9*	*19806*	(*39.7* | *60.3*)
Physically active														
No (< 150 mins pw)	4376	29.2	10632	70.8	*15008*	(25.5 | 74.5)	6340	42.2	8668	57.8	*15008*	(40.8 | 59.2)
Yes (≥ 150 mins pw)	950	19.8	3848	80.2	*4798*	(17.4 | 82.6)	1803	37.6	2995	62.4	*4798*	(36.1 | 63.9)
Totals	*5326*	*26.9*	*14480*	*73.1*	*19806*	(*23.5* | *76.5*)	*8143*	*41.1*	*11663*	*58.9*	*19806*	(*39.7* | *60.3*)
Employed full-time														
No	4355	33.4	8685	66.6	*13040*	(30.9 | 69.1)	5444	41.7	7596	58.3	*13040*	(39.8 | 60.2)
Yes	971	14.4	5795	85.6	*6766*	(14.2 | 85.8)	2699	39.9	4067	60.1	*6766*	(39.5 | 60.5)
Totals	*5326*	*26.9*	*14480*	*73.1*	*19806*	(*23.5* | *76.5*)	*8143*	*41.1*	*11663*	*58.9*	*19806*	(*39.7* | *60.3*)
Married/cohabiting														
No	2767	31.6	5983	68.4	*8750*	(27.7 | 72.3)	4154	47.5	4596	52.5	*8750*	(46.2 | 53.8)
Yes	2559	23.1	8497	76.9	*11056*	(20.5 | 79.5)	3989	36.1	7067	63.9	*11056*	(35.1 | 64.9)
Totals	*5326*	*26.9*	*14480*	*73.1*	*19806*	(*23.5* | *76.5*)	*8143*	*41.1*	*11663*	*58.9*	*19806*	(*39.7* | *60.3*)
Ethnicity – White British														
No	905	19.6	3703	80.4	*4608*	(17.8 | 82.2)	2032	44.1	2576	55.9	*4608*	(43.3 | 56.7)
Yes	4421	29.1	10777	70.9	*15198*	(25.2 | 74.8)	6111	40.2	9087	59.8	*15198*	(38.6 | 61.4)
Totals	*5326*	*26.9*	*14480*	*73.1*	*19806*	(*23.5* | *76.5*)	*8143*	*41.1*	*11663*	*58.9*	*19806*	(*39.7* | *60.3*)
Children in household														
No	4329	30.6	9808	69.4	*14137*	(26.6 | 73.4)	5876	41.6	8261	58.4	*14137*	(40.1 | 59.9)
Yes	997	17.6	4672	82.4	*5669*	(15.4 | 84.6)	2267	40.0	3402	60.0	*5669*	(38.6 | 61.4)

	Totals	*5326*	*26.9*	*14480*	*73.1*	*19806*	(*23.5*	*76.5*)	*8143*	*41.1*	*11663*	*58.9*	*19806*	(*39.7*	*60.3*)
Dog Owner															
No		4058	26.5	11282	73.5	*15340*	(23.1	76.9)	6283	41.0	9057	59.0	*15340*	(39.6	60.4)
Yes		1268	28.4	3198	71.6	*4466*	(24.6	75.4)	1860	41.6	2606	58.4	*4466*	(39.8	60.2)
	Totals	*5326*	*26.9*	*14480*	*73.1*	*19806*	(*23.5*	*76.5*)	*8143*	*41.1*	*11663*	*58.9*	*19806*	(*39.7*	*60.3*)
Year															
2014/2015		2459	26.3	6884	73.7	*9343*	(22.7	77.3)	3888	41.6	5455	58.4	*9343*	(39.8	60.2)
2015/2016		2867	27.4	7596	72.6	*10463*	(24.1	75.9)	4255	40.7	6208	59.3	*10463*	(39.5	60.5)
	Totals	*5326*	*26.9*	*14480*	*73.1*	*19806*	(*23.5*	*76.5*)	*8143*	*41.1*	*11663*	*58.9*	*19806*	(*39.7*	*60.3*)

Note. Weighted %s (in brackets) take into account sample weights.

Appendix C: Relationships between time in nature per week and other covariates in models predicting health and wellbeing.

In order to explore the relationships between our key predictor variable, time spent in nature per week, and other covariates in the models predicting health and wellbeing we split duration into <120 minutes and ≥120 minutes based on the findings discussed elsewhere. The odds ratios (ORs) and 95% confidence intervals (CIs) for the survey weighted binomial logistic regression predicting time spent in nature from the remaining covariates is presented in Supplementary Table S1 below. Results highlight the need to include these variables in our models predicting health and wellbeing because nearly all of them co-varied with time in nature. Specifically, the likelihood of an individual reporting ≥ 120 (vs. <120) minutes last week was higher for individuals: a) who lived in areas with less deprivation and lower air pollution; and b) who were under 65 years old, of higher socio-economic status, without restricted functioning, met weekly physical activity guidelines, were not in full-time employment, whose ethnicity was White-British, had children in the household, and had a dog. Intriguingly, individuals living in the least green quintile were more likely to report ≥ 120mins of recreational contact with nature than individuals living in all four greener quintiles, contrary to a simple, people who live in greener areas are likely to spend more time in nature hypothesis (see also ref. 24 in the main body). The only three variables which did not predict time spent in nature (rurality, sex, year) were retained in the final models for theoretical (e.g. rurality is a key confound of area green space) or statistical (e.g. need to control for survey wave) reasons. Of note, rurality and year were also not significant predictors of health or wellbeing either (though sex was).

Supplementary Table S2. The odds ratios (OR) and 95% confidence intervals (CIs) of reporting ≥120 minutes of nature contact per week as a function of area and individual level variables

	Weekly nature contact (≥120mins vs. <120 mins)		
		95% CIs	
	OR	Low	High
**Area level variables**			
Urbanicity			
Rural	1.07	0.91	1.24
Urban/town fringe	-	-	-
Neighbourhood green			
Greenest quintile	0.80**	0.70	0.91
Quintile 2	0.78***	0.70	0.88
Quintile 3	0.82**	0.73	0.92
Quintile 4	0.78***	0.70	0.88
Least green quintile	-	-	-
Area deprivation			
Least deprived	1.76***	1.56	1.98
Quintile 2	1.57***	1.39	1.76
Quintile 3	1.55***	1.38	1.73
Quintile 4	1.25***	1.12	1.39
Most deprived	-	-	-
Air pollution			
PM_{10} lowest tertile	1.41***	1.29	1.55

PM_{10} middle tertile	1.26***	1.16	1.38
PM_{10} highest tertile	-	-	-
**Individual variables**			
Sex			
Male	-	-	-
Female	0.94	0.87	1.00
Age			
16-64 years	-	-	-
≥ 65 years	0.85***	0.77	0.93
Socio-economic status			
AB (Highest)	1.98***	1.80	2.19
C1	1.69***	1.55	1.85
C2	1.25***	1.14	1.38
DE (Lowest)	-	-	-
Restricted functioning			
No	1.46***	1.34	1.60
Yes	-	-	-
Physically active			
No (< 150 mins pw)	-	-	-
Yes (≥ 150 mins pw)	1.90***	1.76	2.04
Employed full-time			
No	-	-	-
Yes	0.88**	0.82	0.96
Married/cohabiting			
No	-	-	-
Yes	1.14***	1.06	1.22
Ethnicity - White British			
No	-	-	-
Yes	1.44***	1.32	1.58
Children in household			
No	-	-	-
Yes	1.31***	1.21	1.42
Dog Owner			
No	-	-	-
Yes	2.23***	2.06	2.41
Year			
2014/2015	-	-	-
2015/2016	0.94	0.88	1.01
Constant	-2.31	-2.47	-2.16
Pseudo R^2	0.10		
Valid N	19,806		

Note. * $p<0.05$; ** $p<0.01$; *** $p<0.001$

Appendix D: Full details of regression models including all covariates.

Supplementary Table S3. The odds ratios (OR) and 95% confidence intervals (CIs) of reporting good health and high well-being as a function of nature visit duration in the last 7 days

	Self-reported health (Good vs. poor)						Subjective well-being (High vs. low)					
	Unadjusted			Adjusted			Unadjusted			Adjusted		
		95% CIs			95% CIs			95% CIs			95% CIs	
	OR	Low	High	OR	Low	High	OR	Low	High	OR	Low	High
**Nature visit exposure**												
Weekly visit duration												
≥ 300 mins	1.73***	1.57	1.91	1.33***	1.18	1.50	1.42***	1.31	1.54	1.20***	1.09	1.31
240-299 mins	2.10***	1.74	2.53	1.55***	1.25	1.93	1.45***	1.24	1.68	1.25**	1.07	1.46
180-239 mins	1.74***	1.47	2.06	1.44***	1.18	1.76	1.33***	1.16	1.53	1.16*	1.00	1.34
120-179 mins	2.09***	1.79	2.44	1.59***	1.31	1.92	1.37***	1.21	1.55	1.23**	1.08	1.40
60-119 mins	1.56***	1.34	1.83	1.13	0.94	1.37	1.21**	1.06	1.39	1.10	0.96	1.27
1-59 mins	1.14	0.88	1.46	1.04	0.76	1.41	1.05	0.83	1.31	0.99	0.78	1.26
0 mins	ref	ref	ref	ref	ref	ref	ref	ref	ref	ref	ref	ref
**Area level covariates**												
Urbanicity												
Rural	-	-	-	1.01	0.83	1.23	-	-	-	1.06	0.91	1.25
Urban/town fringe	-	-	-	ref	ref	ref	-	-	-	ref	ref	ref
Neighbourhood green												
Greenest quintile	-	-	-	1.05	0.89	1.23	-	-	-	1.15*	1.02	1.30
Quintile 2	-	-	-	0.96	0.83	1.11	-	-	-	0.98	0.88	1.09
Quintile 3	-	-	-	0.97	0.85	1.11	-	-	-	1.03	0.93	1.14
Quintile 4	-	-	-	0.99	0.87	1.13	-	-	-	1.02	0.92	1.13
Least green quintile	-	-	-	ref	ref	ref	-	-	-	ref	ref	ref
Area deprivation												
Least deprived	-	-	-	1.46***	1.25	1.69	-	-	-	1.26***	1.13	1.41
Quintile 2	-	-	-	1.38***	1.20	1.59	-	-	-	1.17**	1.05	1.31

Quintile 3	-	-	-	1.11	.97	1.26	-	-	-	1.18**	1.06	1.30
Quintile 4	-	-	-	1.15*	1.01	1.29	-	-	-	1.07	0.97	1.18
Most deprived	-	-	-	ref	ref	ref	-	-	-	ref	ref	ref
Air pollution												
PM_{10} lowest tertile	-	-	-	0.96	0.86	1.08	-	-	-	1.07	0.98	1.17
PM_{10} middle tertile	-	-	-	1.02	0.92	1.14	-	-	-	1.03	0.95	1.12
PM_{10} highest tertile	-	-	-	ref	ref	ref	-	-	-	ref	ref	ref
**Individual covariates**												
Sex												
Male	-	-	-	ref	ref	ref	-	-	-	ref	ref	ref
Female	-	-	-	1.18***	1.08	1.28	-	-	-	1.16***	1.09	1.24
Age												
16-64 years	-	-	-	ref	ref	ref	-	-	-	ref	ref	ref
≥ 65 years	-	-	-	0.95	0.85	1.06	-	-	-	1.55***	1.42	1.69
Socio-economic status												
AB (Highest)	-	-	-	1.52***	1.34	1.73	-	-	-	1.40***	1.27	1.54
C1	-	-	-	1.40***	1.26	1.55	-	-	-	1.23***	1.13	1.34
C2	-	-	-	1.30***	1.16	1.45	-	-	-	1.14**	1.05	1.24
DE (Lowest)	-	-	-	ref	ref	ref	-	-	-	ref	ref	ref
Restricted functioning												
No	-	-	-	11.55***	10.56	12.64	-	-	-	2.00***	1.84	2.16
Yes	-	-	-	ref	ref	ref	-	-	-	ref	ref	ref
Physically active												
No (< 150 mins pw)	-	-	-	ref	ref	ref	-	-	-	ref	ref	ref
Yes (≥ 150 mins pw)	-	-	-	1.43***	1.29	1.59	-	-	-	1.17***	1.08	1.26
Employed full-time												
No	-	-	-	ref	ref	ref	-	-	-	ref	ref	ref
Yes	-	-	-	1.49***	1.34	1.65	-	-	-	0.96	0.89	1.03
Married/cohabiting												
No	-	-	-	ref	ref	ref	-	-	-	ref	ref	ref
Yes	-	-	-	1.05	0.96	1.15	-	-	-	1.45***	1.37	1.57

Ethnicity - White British												
No	-	-	-	ref	ref	ref	-	-	-	ref	ref	ref
Yes	-	-	-	0.81***	0.72	0.91	-	-	-	1.07	0.98	1.16
Children in household												
No	-	-	-	ref	ref	ref	-	-	-	ref	ref	ref
Yes	-	-	-	1.26***	1.14	1.40	-	-	-	1.01	0.93	1.09
Dog Owner												
No	-	-	-	ref	ref	ref	-	-	-	ref	ref	ref
Yes	-	-	-	0.85**	0.77	0.94	-	-	-	0.92*	0.85	1.00
Year												
2014/2015	-	-	-	ref	ref	ref	-	-	-	ref	ref	ref
2015/2016	-	-	-	0.95	0.87	1.03	-	-	-	1.02	0.96	1.09
Constant	2.61	2.50	2.72	0.28	0.24	0.33	1.34	1.29	1.39	0.36	0.31	0.41
Pseudo R^2	0.01			0.23			0.01			0.05		
Valid N	19,806			19,806			19,806			19,806		

Note. * p<0.05; ** p<0.01; ***p<0.001

Appendix E: Sensitivity analyses modelling outcomes as ordinal and linear variables

We examined the sensitivity of the models to collapsing the outcome data into binary variables by running ordered logistic models, adjusted for covariates, with the full outcome data. Note that Stata does not allow a test of the proportional odds assumption with sample weighted data, and this is a further reason for our preference for logistic regression models.

Supplementary Table S4: Binary and Ordered logistic outcome of the 5 point General Health outcome variable (adjusted model).

	Self-reported health (Good vs. poor)			Self-reported health (Ordinal)		
	OR	95% CIs		OR	95% CIs	
		Low	High		Low	High
**Nature visit exposure**						
Weekly visit duration						
≥ 300 mins	1.33***	1.18	1.50	1.25***	1.16	1.36
240-299 mins	1.55***	1.25	1.93	1.44***	1.26	1.66
180-239 mins	1.44***	1.18	1.76	1.39***	1.22	1.59
120-179 mins	1.59***	1.31	1.92	1.31***	1.17	1.47
60-119 mins	1.13	0.94	1.37	1.14*	1.00	1.29
1-59 mins	1.04	0.76	1.41	1.20	0.95	1.51
0 mins	ref	ref	ref	ref	ref	ref

Notes. Results controlling for: urbanicity, neighbourhood greenspace, area deprivation, background air pollution, sex, age, socioeconomic status, restricted physical functioning, physical activity, employment status, relationship status, ethnicity, children in household, dog ownership and year. * $p<0.05$; ** $p<0.01$; *** $p<0.001$

Although the interpretations of OR differ in the logistic and ordinal regressions, the main patterns observed in the (collapsed) binary logistic outcome for General Health in the main paper are repeated in the ordinal regression: a) exposure of 1-59 minutes showed no significant increased likelihood of reporting (binary outcome) good health, and it showed no significant increased likelihood of reporting (ordinal outcome) better health; b) though the coefficients for exposure of 60-119 mins are almost the same in the binary and ordinal models, (1.13/1.14), this increased likelihood of reporting good health in the binary model was not significant, but this increased likelihood of reporting better health in the ordinal model is significant at $p < 0.05$ (0.043); c) we see a comparable step change in the increased likelihood of both good health in the binary model and better health in the ordinal model for exposure at 120-179; 180-239 and 240-299 mins (compared to at exposure at 1-59 and 60-

119 mins), with OR at these three levels similar to one another in both cases, and significant at p<0.001 in both cases; d) in both the binary and ordinal models we see some decline in the effect at exposures ≥ 300 mins, which is similar to the linear models in Figure 2.

Supplementary Table S5: Binary and Ordered logistic outcome of the 11 point Wellbeing outcome variable (adjusted model).

	Subjective wellbeing (High vs. low)			Subjective wellbeing (Ordinal)		
	OR	95% CIs		OR	95% CIs	
		Low	High		Low	High
Weekly visit duration						
≥ 300 mins	1.20***	1.09	1.31	1.19***	1.11	1.25
240-299 mins	1.25**	1.07	1.46	1.16*	1.03	1.31
180-239 mins	1.16*	1.00	1.34	1.22**	1.09	1.36
120-179 mins	1.23**	1.08	1.40	1.19**	1.08	1.31
60-119 mins	1.10	0.96	1.27	1.12*	1.01	1.25
1-59 mins	0.99	0.78	1.26	0.92	0.75	1.13
0 mins	ref	ref	ref	ref	ref	ref

Notes. Results controlling for: urbanicity, neighbourhood greenspace, area deprivation, background air pollution, sex, age, socioeconomic status, restricted physical functioning, physical activity, employment status, relationship status, ethnicity, children in household, dog ownership and year. * p<0.05; ** p<0.01; ***p<0.001

The main patterns observed in the (collapsed) binary logistic outcome for Wellbeing in the submission are repeated in the ordinal regression: a) exposure of 1-59 minutes showed no significant increased likelihood of reporting (binary outcome) high wellbeing, and it shows no significant increased likelihood of reporting (ordinal outcome) better wellbeing; b) though the coefficients for exposure of 60-119 mins are almost the same in the binary and ordinal models, (1.1/1.12), this increased likelihood of reporting good health in the binary model was not significant, but this increased likelihood of reporting better health in the ordinal model is significant at p <0.05 (0.038); c) we see a comparable step change in the increased likelihood of both high wellbeing in the binary model and better wellbeing in the ordinal model for exposure at 120-179; 180-239; 240-299 mins and ≥ 300 mins, with OR at these three levels similar to one another in both cases, and significant in both cases.

Figure D: The relationship between duration in nature and wellbeing with both variables modelled linearly (note the extremely high confidence intervals beyond ~400 minutes).

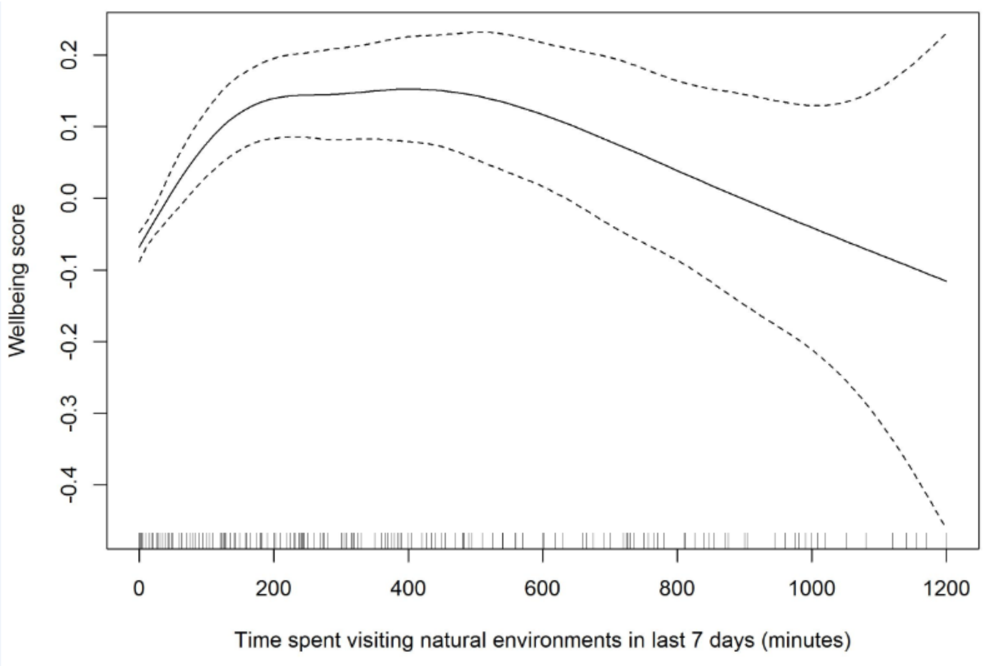

Appendix F: The frequency and percent of respondents in each category of derived binary variables used in stratified analyses.

Supplementary Table S6: The frequency and percent of respondents in each category of derived binary variables used in stratified analyses, and of respondents in each of the ≥120 mins duration composition categories, who reported good/very good health and high well-being.

	Self-reported health							Subjective well-being (Life satisfaction)						
	Raw Ns and %s					(Weighted %s)		Raw Ns and %s					(Weighted %s)	
	Not good		Good			Not good	Good	Low		High			Low	High
	N	%	N	%	Total N	%	%	N	%	N	%	Total N	%	%
Weekly visit duration														
≥120 mins	1298	19.5	5372	80.5	*6670*	(17.3	82.7)	2376	35.6	4294	64.4	*6670*	(34.8	65.2)
1-119 mins	350	23.8	1118	76.2	*1468*	(21.0	79.0)	594	40.5	874	59.5	*1468*	(39.0	61.0)
0 mins	3687	31.5	7990	38.5	*11668*	(27.7	72.3)	5173	44.3	6495	55.7	*11668*	(42.8	57.2)
Totals	*5326*	*26.9*	*14480*	*73.1*	*19806*	(*23.5*	*76.5*)	*8143*	*41.1*	*11663*	*58.9*	*19806*	(*39.7*	*60.3*)
Neighbourhood green														
High	2292	26.9	6218	73.1	*8510*	(23.6	76.4)	3231	38.0	5279	62.0	*8510*	(36.5	63.5)
Low	3034	26.9	8262	73.1	*11296*	(23.4	76.6)	4912	43.5	6384	56.5	*11296*	(42.4	57.8)
Totals	*5326*	*26.9*	*14480*	*73.1*	*19806*	(*23.5*	*76.5*)	*8143*	*41.1*	*11663*	*58.9*	*19806*	(*39.7*	*60.3*)
Area Deprivation														
High	3516	29.8	8280	70.2	*11796*	(26.1	73.9)	5276	44.7	6520	55.3	*11796*	(43.4	56.6)
Low	1810	22.6	6200	77.4	*8010*	(20.1	79.9)	2867	35.8	5143	64.2	*8010*	(34.9	65.1)
Totals	*5326*	*26.9*	*14480*	*73.1*	*19806*	(*23.5*	*76.5*)	*8143*	*41.1*	*11663*	*58.9*	*19806*	(*39.7*	*60.3*)
Socio-economic status														
High	1653	19.2	6971	80.8	*8624*	(18.6	81.4)	3093	35.9	5531	64.1	*8624*	(35.7	64.3)
Low	3673	32.8	7509	67.2	*11182*	(29.2	70.8)	5050	45.2	6132	54.8	*11182*	(44.3	55.7)
Totals	*5326*	*26.9*	*14480*	*73.1*	*19806*	(*23.5*	*76.5*)	*8143*	*41.1*	*11663*	*58.9*	*19806*	(*39.7*	*60.3*)
**Visit frequency**														
≥120mins per week only														
1 visit ≥ 120mins	420	18.8	1810	81.2	*2230*	(16.4	83.6)	800	35.9	1430	64.1	*2230*	(35.5	64.5)

2 visits ≥ 60 ≤119mins	67	17.5	316	82.5	*383*	(16.3	83.7)	132	34.5	251	65.5	*383*	(35.0	65.0)
≥3 visits ≤40mins	174	22.8	588	77.2	*762*	(20.3	79.7)	280	36.7	482	63.3%	*762*	(35.6	64.5)
Totals	661	19.6	2714	80.4	*3375*	(17.3	82.7)	1212	35.9	2163	64.1%	*3375*	(35.5	64.5)

Note. Weighted %s (in brackets) take into account sample weights.

Appendix G: Does the way in which ≥120mins in nature per week is attained matter for health and well-being outcomes?

To explore whether the manner in which 'high contact' ≥120mins (i.e. ≥120mins) was achieved might be important we focused only on those who reported ≥120mins and constructed three exposure/contact groups: a) those who achieved this level in a single visit (≥120mins); those who achieved it in two visits (≥ 60-≤119mins), and those who achieved it in ≥three visits (≥40mins =*ref*). More categories were not possible with the reduced sample, and results were similar if we restricted the sample to only those who reported 120-179mins or all respondents ≥120mins. Supplementary Table S7 presents the results below. Although the odds of good health were significantly higher for those who achieved the threshold on just one vs. ≥three visits in the unadjusted model, this effect disappeared in the fully adjusted model. There were no differences in either model for well-being. In short, the odds of reporting positive health and well-being outcomes were unrelated to how the threshold of weekly nature contact was achieved. Preliminary analysis (not presented) also found no effect of whether the visits involved active (e.g. jogging) or inactive (e.g. enjoying the view from a car) activities. Moreover, it was also not possible to see whether nature visit companions (e.g. alone, other adults, children) were important because companion data were not collected in the same sampling frame as the health and well-being outcomes. Given the potential importance of social connectedness from nature visits (Kaźmierczak, 2013; Sugiyama, Leslie, Giles-Corti, & Owen, 2008; Weinstein, Balmford, DeHaan, et al., 2015) this is something else that will need to be considered in relation to exposure thresholds going forward.

References

Kaźmierczak, A. (2013). The contribution of local parks to neighbourhood social ties. *Landscape and Urban Planning*, *109*(1), 31-44

Sugiyama, T., Leslie, E., Giles-Corti, B., & Owen, N. (2008). Associations of neighbourhood greenness with physical and mental health: do walking, social coherence and local social interaction explain the relationships?. *Journal of Epidemiology and Community Health*, *62*(5), e9-e9.

Weinstein, N., Balmford, A., DeHaan, C. R., Gladwell, V., Bradbury, R. B., & Amano, T. (2015). Seeing community for the trees: the links among contact with natural environments, community cohesion, and crime. *BioScience*, *65*(12), 1141-1153.

Supplementary Table S7: The odds ratios (OR) and 95% confidence intervals (CIs) of reporting good health and high well-being among those who visited nature ≥ 120mins in the last 7 days as a function of visit distribution

	Self-reported health (Good vs. poor)						Subjective well-being (High vs. low)					
	Unadjusted			Adjusted[a]			Unadjusted			Adjusted[a]		
		95% CIs			95% CIs			95% CIs			95% CIs	
	OR	Low	High	OR	Low	High	OR	Low	High	OR	Low	High
**Visit distribution**												
≥120mins per week												
1 visit ≥ 120mins	1.30**	1.05	1.61	1.07	0.81	1.42	1.00	0.84	1.20	1.01	0.81	1.25
2 visits ≥ 60 ≤119mins	1.30	0.94	1.82	0.96	0.65	1.44	1.02	0.78	1.34	0.97	0.73	1.30
≥3 visits ≤40mins	ref	ref	ref	ref	ref	ref	ref	ref	ref	ref	ref	ref
Covariates												
Area	NO			NO			NO			NO		
Individual	NO			YES			NO			YES		
Constant	3.93			0.40			1.81			0.57		
Pseudo R^2	.00			.16			.00			.04		
Valid N	3,375			3,375			3,375			3,375		

Note. [a]Full models available on request.
